# Exosomes multiplex profiling, a promising strategy for early diagnosis of laryngeal cancer

**DOI:** 10.1186/s12967-024-05396-0

**Published:** 2024-06-20

**Authors:** Marco Bocchetti, Amalia Luce, Clara Iannarone, Lucia Stefania Pasquale, Michela Falco, Chiara Tammaro, Marianna Abate, Maria Grazia Ferraro, Raffaele Addeo, Filippo Ricciardiello, Giovanni Motta, Luca De Stefano, Francesco Caraglia, Anna Ceccarelli, Silvia Zappavigna, Marianna Scrima, Alessia Maria Cossu, Michele Caraglia, Gabriella Misso

**Affiliations:** 1 Precision Medicine Department, University of Campania “Luigi Vanvitelli”, Via De Crecchio, Naples, 80131 NA Italy; 2 Molecular Oncology and Precision Medicine Laboratory, Biogem Scarl, Contrada Camporeale, Ariano Irpino, 83031 AV Italy; 3 Molecular Medicine and Medical Biotechnology Department, University of Naples “Federico II”, Via Pansini, 5, Naples, 80131 NA Italy; 4 Oncology Unit, “S. Giovanni di Dio” Hospital, Via Pirozzi, Frattamaggiore, 80020 NA Italy; 5 ORL Complex Operative Unit, AORN “Cardarelli”, Via Antonio Cardarelli, 9, Naples, 80131 NA Italy; 6 Institute of Applied Sciences and Intelligent Systems, National Research Council (CNR), Via P. Castellino, 111, Naples, 80131 NA Italy; 7 Oncohematology Complex Operative Unit, University of Campania “Luigi Vanvitelli”, Via Sergio Pansini, Naples, 80131 NA Italy; 8 Medical Oncology, Catholic University of the Sacred Heart, Rome, 00168 RM Italy; 9 Nephrology Complex Operative Unit, Translational Medicine Department, University of Campania “Luigi Vanvitelli”, Via De Crecchio, Naples, 80131 NA Italy

**Keywords:** Exosomes, Laryngeal cancer, Membrane epitopes, Early diagnosis, Liquid biopsy

## Abstract

**Background:**

Exosomes are nanosized vesicles released from all cells into surrounding biofluids, including cancer cells, and represent a very promising direction in terms of minimally invasive approaches to early disease detection. They carry tumor-specific biological contents such as DNA, RNA, proteins, lipids, and sugars, as well as surface molecules that are able to pinpoint the cellular source. By the above criteria, exosomes may be stratified according to the presence of tissue and disease-specific signatures and, due to their stability in such biofluids as plasma and serum, they represent an indispensable source of vital clinical insights from liquid biopsies, even at the earliest stages of cancer. Therefore, our work aimed to isolate and characterize LCa patients’ derived exosomes from serum by Flow Cytometry in order to define a specific epitope signature exploitable for early diagnosis.

**Methods:**

Circulating exosomes were collected from serum collected from 30 LCa patients and 20 healthy volunteers by the use of antibody affinity method exploiting CD63 specific surface marker. Membrane epitopes were then characterized by Flow cytometry multiplex analysis and compared between LCa Patients and Healthy donors. Clinical data were also matched to obtain statistical correlation.

**Results:**

A distinct overexpression of CD1c, CD2, CD3, CD4, CD11c, CD14, CD20, CD44, CD56, CD105, CD146, and CD209 was identified in LCa patients compared to healthy controls, correlating positively with tumor presence. Conversely, CD24, CD31, and CD40, though not overexpressed in tumor samples, showed a significant correlation with nodal involvement in LCa patients (p < 0.01).

**Conclusion:**

This approach could allow us to set up a cost-effective and less invasive liquid biopsy protocol from a simple blood collection in order to early diagnose LCa and improve patients’ outcomes and quality of life.

**Graphical Abstract:**

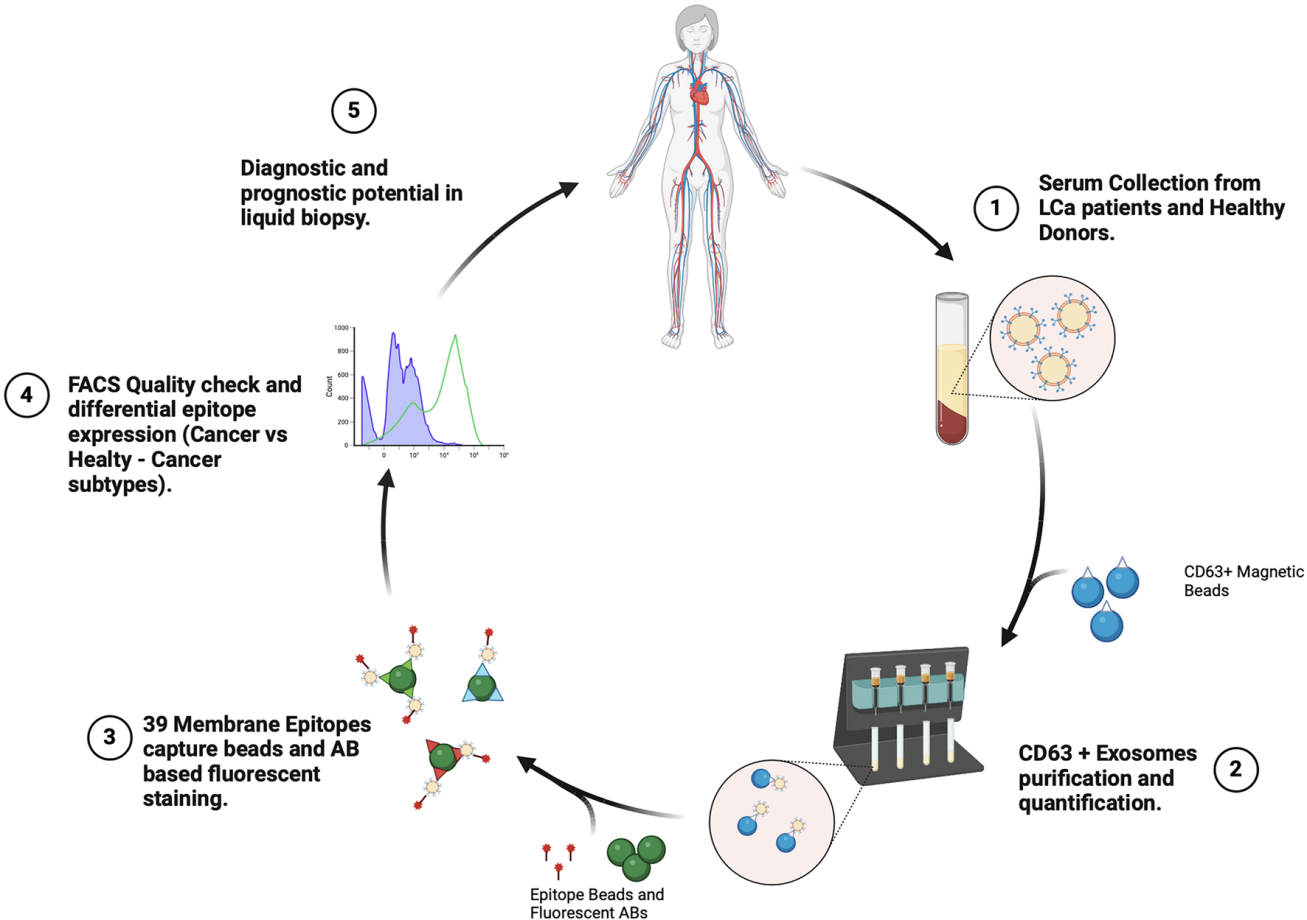

## Background

Exosomes are small vesicles (30–100 nanometers) originating from the cells by different biological processes [1]. Those vesicles are present in almost all body fluids and perform central functions in a number of biological roles like intercellular communication, immune responses, and elimination of cellular waste [2]. A strong relationship with cancer progression is evidently pointed out by the mere fact that these vehicles are the ones transferring from the primary location of the tumor to other parts of the body [3, 4]. This makes them a focal point in research for potential use as organ-specific markers especially in liquid biopsies [5]. Due to their diverse molecular composition and the ability of their surface proteins to reflect their tissue of origin, exosomes represent a unique opportunity to identify those specific diseases [6–8]. In this light, it could be crucial to use those incredibly useful nanovescicles for early cancer diagnosis, especially for hardly detectable malignancies such as Laryngeal Cancer (LCa) [9]. LCa accounts for almost one-third of head and neck cancers and is the sixth most common cancer worldwide [10]. The main risk factors associated with LCa are tobacco and alcohol abuse together with HPV infections (especially HPV16)[11]. Diagnosis is based on neck palpation in the first place, followed by CT or MRI and PET. To date, depending on the stadiation and grading of the tumor a dedicated multidisciplinary team (MDT) assesses the correct treatment choices, starting from CO2 laser excision and/or radiotherapy for T1 and T2 or surgery combined with chemotherapy and immunotherapy [12]. Total laryngectomy and neck dissection are reserved for patients with extensive and advanced tumors, with lymph node metastases [12]. Onset varies between different larynx parts, and every one of those shows different symptoms. The survival rate ranges around 60% and it is deeply influenced by the late diagnoses (together with the clinical approach and the invasiveness of surgery, reflecting the subsequent patients quality of life). Currently, early diagnosis eludes two-thirds of LCa patients, primarily due to the scarcity of effective diagnostic biomarkers. Although a large number of diagnostic and prognostic biomarkers [10, 13, 14] have been proposed over the last few decades for diagnosis and prognosis, barely a fraction have been incorporated into clinical settings yet. The gap from development to clinical use underscores that the biomarkers usually do not meet the essential requirements of high specificity and sensitivity, affordability, high positive predictive values, substantial clinical significance, and rapid turnaround. The complexity of LCa additionally complicates this issue, as their significant heterogeneity and also anatomical site of origin cause that no single biomarker can offer optimal sensitivity. Nevertheless, using a combination of biomarkers or signatures can substantially improve the power of the marker for diagnostic and prognostic purposes by providing a holistic view through the molecular analysis of diverse liquid biopsy samples from an individual patient. This is exactly the reason why we started, and our work aimed to analyze and characterize exosome surface membrane proteins from LCa patients’ serum. Over the past 7 years, serum samples have been collected from patients with LCa across various medical centers in Campania, including the “A. Cardarelli” Hospital, the University of Campania “L. Vanvitelli”, Federico II University, and the “V. Monaldi” Hospital. Additionally, serum samples from healthy volunteers have also been compiled. These samples, complemented by patients’ clinical data such as tumor stage, nodal involvement and metastatization were stored at − 80 °C. Among these, 30 of the most recently obtained serum samples from patients and 20 from healthy individuals were selected to match the analysis’ sample size. Exosomal vesicles from LCa patients and healthy subjects were isolated and subsequently characterized using immunoaffinity techniques that use antigen-antibody interactions in order to understand how to use those markers to enhance early detection of LCa by liquid biopsy approaches.

## Methods

### Ethical statements and serum storage

The prospective study was approved by in accordance with the Institutional Ethics Committee guidelines, Italian law and the Declaration of Helsinki, and was approved by the Ethics Committee of University of Campania “Luigi Vanvitelli” - Azienda Ospedaliera Universitaria “Luigi Vanvitelli” - AORN “Ospedali dei Colli” (Approval number: 25445/2021). All participants provided informed consent before their inclusion. Regarding the data showcased in this research, the procedure for processing serum samples was as follows: Blood samples, each 5 mL, both from healthy volunteers and LCa patients were collected using heparin and serum-separating tubes. The inclusion criteria for LCa patients involved a confirmed histopathological diagnosis, with no restrictions on disease stage to capture a comprehensive range of cancer progression. Exclusion criteria included prior chemotherapy or radiotherapy, ensuring that the exosomal profile was not influenced by treatment.

### Dataset description

Our patients’ cohort was composed of 30 LCa patients, ranging from early to advanced stages of the disease, and 20 age-matched healthy volunteers. The LCa patients included various subtypes of laryngeal carcinoma, reflecting the heterogeneity of this cancer type. Patients’ data including sex, age, TNM, Grade, alcohol and smoke abuse were collected, providing a robust foundation for correlating exosomal epitope expressions with disease characteristics. In detail, our patients were mainly males (27 out of 30), with ages ranging from 51 to 74 years, with a median age of 60 years. Regarding TNM, 2 out of 30 had a T1 clinical/pathological scoring, 8 out of 30 T2, 9 out of 30 T3 and 9 out of 30 T4. 15 out of 30 showed no lymph node involvement while 2 out of 30 had N1, 9 out of 30 N2 and 2 out of 30 N3 involvement at pathological staging. 4 patients out of 30 experienced a relapse, while only 3 patients experienced metastasis (Table 1).

**Table 1:** Dataset description

Sex	Female — 3 (10%)	Male — 27 (90%)		
Age	Min — 51	Max — 74	Median — 60	
T	T1 — 2 (7%)	T2 — 8 (29%)	T3 — 9 (32%)	T4 — 9 (32%)
N	N0 — 15 (54%)	N1 — 2 (7%)	N2 — 9 (32%)	N3 — 2 (7%)
M	3			
Relapse	4			

Brief description of the patients’ cohort

### Exosomes extraction from patients’ serum

The isolation of exosomes is performed by positive selection using MicroBeads recognizing the tetraspanin proteins CD63 using The Exosome Isolation Kit (Miltenyi Biotec, Bergisch-Gladbach, Germany) according to the manufacturer’s protocol. Briefly, EVs were magnetically labeled during a short incubation period. The labeled EVs were loaded onto a $\upmu$ Column (Miltenyi Biotec, Bergisch-Gladbach, Germany), which was placed in the magnetic field of a $\upmu$MACS$^{\textrm{TM}}$ Separator (Miltenyi Biotec, Bergisch-Gladbach, Germany). The magnetically labeled EVs were retained within the column, while the unlabeled vesicles and cell components ran through the column. After removing the column from the magnetic field, the intact EVs were collected by elution with Isolation Buffer. Serum samples from both LCa patients and healthy volunteers (1 mL volume, diluted 1:1 with PBS) were incubated with a buffer containing CD63 binding beads. This choice was motivated by CD63’s abundant expression on the exosomal surface, facilitating efficient capture and isolation. The process was optimized to enhance yield and purity. Portions of the eluted exosomes were then quantified for comparative characterization across samples from both groups.

### Exosomes quantification

Eluted exosome fraction was quantified by global protein content to uniform the input between all samples for subsequent epitope characterization. Briefly, we lysed (Lysis buffer composition:) 20 μL of the exosome fraction and quantified the protein absorbance using Bio-Rad Protein Assay (Bio-Rad Laboratories, Inc., California, USA) through spectrometer at 595 nm wavelength. We proceeded to compare obtained protein values with a fixed concentration BSA Standard curve (Thermo Fisher Scientific, Massachusetts, USA) and we took into account the Lysis buffer intrinsic absorbance by subtracting it from the final value obtained.

### Exosomes epitopes multiplex profiling by flow cytometry

The surface protein expression of the exosomes was determined using flow cytometry (BD FACSMelody) with the MACSPlex Exosome human kit (Miltenyi Biotec, Bergisch-Gladbach, Germany). The kit was used for the simultaneous detection and characterization of 37 known exosomal surface epitopes (and control markers) and it was used according to the manufacturer’s protocol. Briefly, we added 200 μL of MACSPlex Buffer in every kit provided plate well we intended to use, plus one designed for the blank for the initial equilibration of the membranes. Subsequently, we removed the buffer by aspiration from the bottom of the well using a void pump. Subsequently, we put the same amount of serum-derived exosomes in the wells reaching the desired final volume (120 μL) topping with the buffer, while only plan buffer was used for the blank sample. The plate was incubated overnight on an orbital shaker at RT (450 RT). After the incubation, 200 μL of buffer was added to each well and then removed by bottom aspiration from each well. Subsequently, we added 135 μL buffer and 15 μL APC fluorescent antibody cocktail (CD9 + CD81 since CD63 was saturated due to the isolation kit) to all used wells including the blank and incubated for 1 h at RT on an orbital shaker (450 RPM). Flow cytometry gating strategy was set manually for every epitope by the use of setup beads reading, excluding doublets and reading artifacts or debris. Every serum sample was extracted, processed and red twice in order to get technical replicates for both healthy donors and LCa patients obtaining more than 100 readings in total. Setup beads reading was recorded during every different flow cytometry run to assure consistency between different readings.

### Flow cytometry and statistical data analysis

Flow cytometry raw data were processed using FlowJo software version 10 in order to obtain mean fluorescence relative to every epitope abundance for each sample read. Subsequently, the mean fluorescence recorded was normalized on blank reading and then by internal experiment controls. Those normalizations were handled by Excel and plotted by the use of GraphPad PRISM ver.9, together with the t-test analyses when comparing healthy and tumor samples. Clinical information datasets, bivariate correlations and ROC curves, together with the model quality plots indicating a graphical representation of the lower bound of the confidence interval of the calculated ROC Area Under the Curve (AUC) were carried out using SPSS ver. 29. In order to obtain the combined ROC curves, we built a predictive model exploiting binary logistic regression in SPSS environment. With this method, we incorporated more than one variable (covariates) in a single calculated predictor for the event to occur (tumor presence or nodal involvement). The prediction probability coefficients obtained in this way for the two signatures were used as a test variable to build the combined ROC curves. Elastic Network model analysis was carried out using R with glmnet, pROC, readx1 and caret packages.

## Results

### Profiling of exosomal surface epitopes

**Fig. 1:**
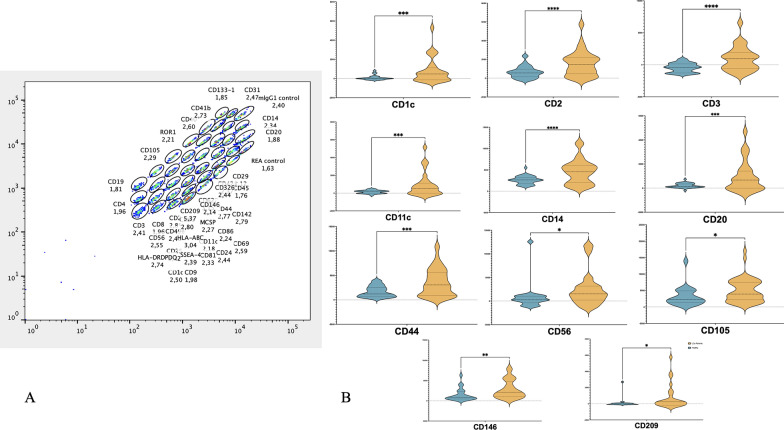
**A** Example of multiplex profiling of every epitope abundance by beads population differential fluorescence **B** Violin plots of the specific epitope normalized MFI in LCa patients (Yellow) compared to healthy donors (Blue) including t-test, * p-value<0.05, ** p-value<0.01, *** p-value<0.001, **** p-value<0.0001

The core of our investigation focused on the multiplex profiling of exosomal surface epitopes through flow cytometry. Remarkably, we identified a distinct overexpression of CD1c, CD2, CD3, CD4, CD11c, CD14, CD20, CD44, CD56, CD105, CD146, and CD209 on exosomes derived from LCa patients. This distinctive overexpression pattern forms a biomarker signature indicative of LCa presence, differentiating it from the healthy control group. Notably, the mean fluorescence intensity (MFI) normalized using technical and biological controls for these markers was higher in the LCa group, showing the least p-values<0.05 (t-test) for each epitope, underlining their potential as biomarkers for LCa. Figure 1 describes the output (A) and underlines the specific epitope amount in LCa patients in comparison with the healthy donor group (Violin plots, Panel B).

### Correlation with clinical features

A deeper analysis into the clinical relevance of these markers showed a significant correlation between their expression levels with both tumor presence and, interestingly lymph node involvement, as well. Pearson’s correlation analysis underscored the direct association of CD1c, CD2, CD3, CD4, CD11c, CD14, CD20, CD44, CD56, CD63, CD69, CD86, CD105 and CD146 levels to the presence of the tumor, while CD24, CD31, and CD40 epitopes showed a significant positive correlation with nodal involvement (p < 0.01), despite not being overexpressed specifically in tumor samples compared to healthy ones. This suggests their potential role in assessing disease severity and progression. Tables 2 and 3 summarize all the statistically significant correlations found, including the coefficient and the p-value.

**Table 2:** Pearson correlation with cancer presence

Epitope	Correlation coefficient	p-value (2-tailed)	N
CD2	0.244	0.015	99
CD3	0.222	0.027	99
CD11c	0.298	0.003	99
CD14	0.395	< 0.001	99
CD20	0.215	0.033	99
CD44	0.240	0.017	99
CD56	0.363	< 0.001	99
CD63	0.215	0.033	99
CD69	0.276	0.006	99
CD86	0.324	0.032	99
CD105	0.245	0.014	99
CD146	0.223	0.026	99

In this table we gathered all the epitopes whose expression is significantly correlating with the cancer presence

**Table 3:** Pearson correlation with nodal involvement

Epitope	Correlation coefficient	p-value (2-tailed)	N
CD24	0.360	0.009	52
CD31	0.365	0.008	52
CD40	0.475	< 0.001	52

In this table we gathered all the epitopes whose expression is significantly correlating with the nodal involvement

To enhance the statistical relevance of the correlation we also applied Spearman correlation analysis which is less susceptible to outliers and gives us an idea of the monotone interaction between variables. In Tables 4 and 5 we report significant Spearman coefficients and p values corroborating the previously found bivariate correlation.

**Table 4:** Spearman correlation with cancer presence

Epitope	Correlation coefficient	p-value (2-tailed)	N
CD2	0.383	< 0.001	99
CD3	0.349	< 0.001	99
CD11c	0.277	0.005	99
CD14	0.330	< 0.001	99
CD20	0.249	0.013	99
CD44	0.328	< 0.001	99
CD56	0.395	< 0.001	99
CD63	0.216	0.032	99
CD69	0.273	0.006	99
CD86	0.324	0.032	99
CD105	0.314	0.002	99
CD146	0.368	< 0.001	99

In this table we gathered all the epitopes showing significant Pearson’s correlation with cancer presence

**Table 5:** Spearman correlation with nodal involvement

Epitope	Correlation coefficient	p-value (2-tailed)	N
CD24	0.264	0.05	52
CD31	0.290	0.037	52
CD40	0.435	< 0.001	52

In this table we gathered all the epitopes showing significant Pearson’s correlation with nodal involvement

### Diagnostic and prognostic value of exosomes expression

**Fig. 2:**
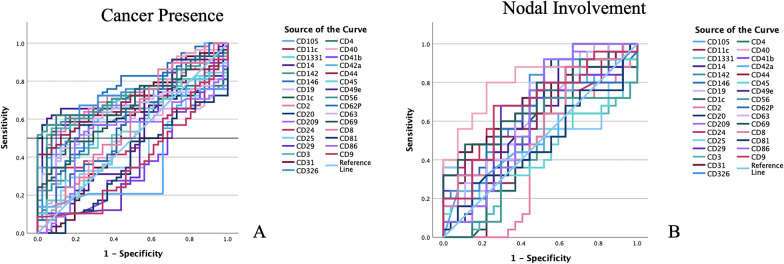
**A** Epitopes ROC curves AUCs related to tumor presence. **B** Epitopes ROC curves AUCs related to Nodal Involvement

**Fig. 3:**
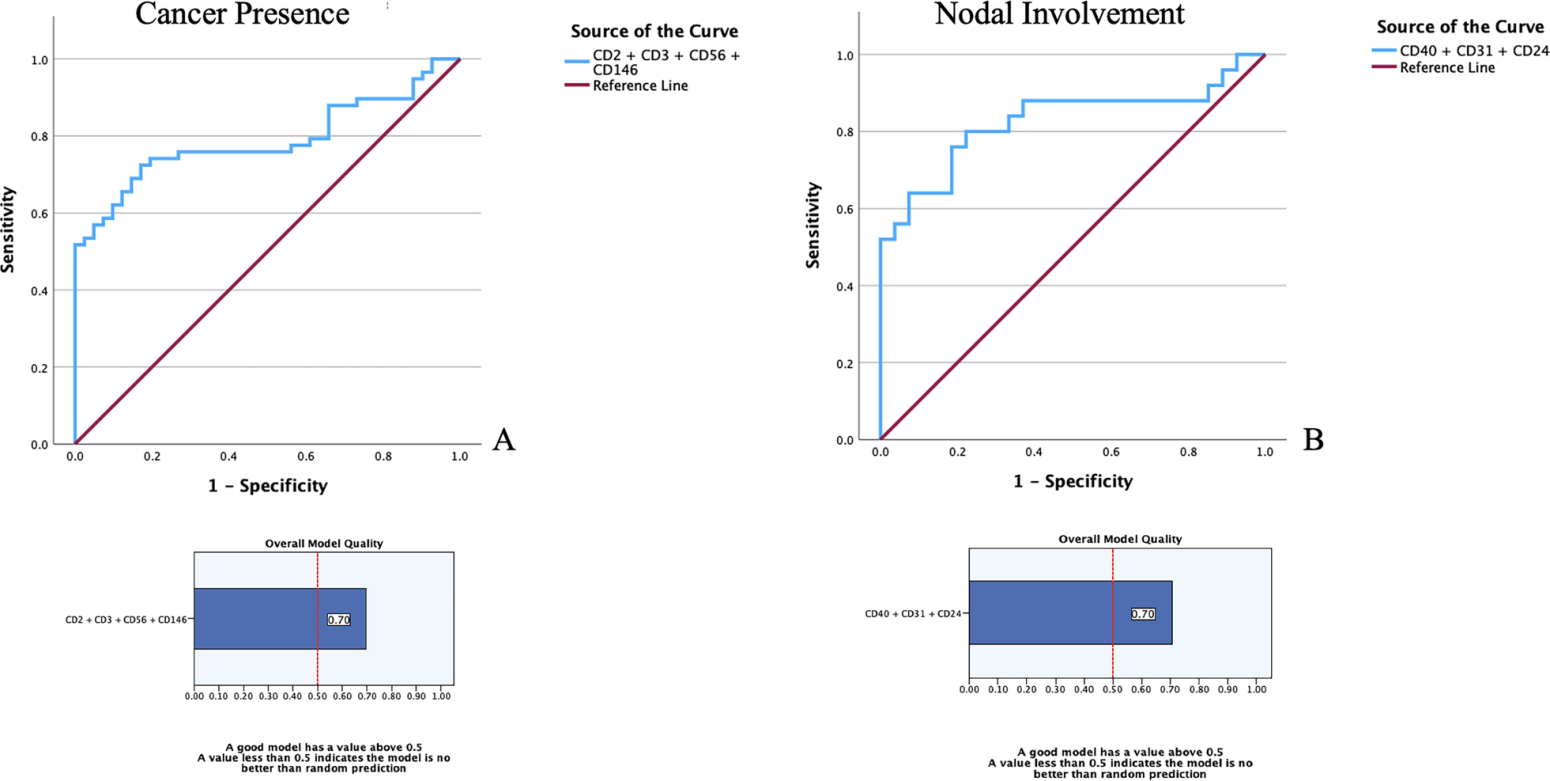
**A** Combined ROC curve including the four most promising epitopes in order to obtain a combined diagnostic signature (TPS). **B** Combined ROC curve including the three most promising epitopes in order to obtain a combined prognostic signature (NIS). The model quality plots are graphical representation of the lower bound of the confidence interval of the Area Under the Curve (AUC)

Receiver Operating Characteristic (ROC) curves were designed to obtain the specific prediction value of every single epitope regarding the tumor presence and nodal involvement. Each epitope AUC, representing a cumulative sensitivity and specificity prediction value for LCa occurrence, is reported in Table 6. In Fig. 2A, curves correlated to tumor diagnosis are shown, while in Table 7 and Fig. 2B those related to nodal involvement. Subsequently, the most promising epitopes were combined to obtain signature-specific ROC curves, as described in the Methods section, both for the TPS (tumor presence signature: CD2, CD3, CD56, CD146) and the NIS (Nodal Involvement Signature: CD24, CD31, CD40) showing combined AUCs which are higher compared to the single epitopes’ AUC. In particular, the AUC values for TPS and NIS were 0.788 and 0.827, respectively, as reported in Fig. 3. The overall model quality was 0.7 out of 1.0, indicating these signatures as good predictor patterns.

**Table 6:** ROC AUC score—Epitopes’ expression and cancer occurrence

Epitope	AUC	Epitope	AUC	Epitope	AUC
CD1c	0.617	CD25	0.459	CD63	0.627
CD2	0.725	CD29	0.453	CD69	0.660
CD3	0.704	CD31	0.549	CD81	0.375
CD4	0.666	CD40	0.556	CD86	0.522
CD8	0.577	CD41b	0.378	CD105	0.684
CD9	0.394	CD42a	0.368	CD133	0.618
CD11c	0.663	CD44	0.692	CD142	0.587
CD14	0.693	CD45	0.518	CD146	0.716
CD19	0.596	CD49	0.508	CD209	0.649
CD20	0.646	CD56	0.731	CD326	0.688
CD24	0.500	CD62	0.592

**Table 7:** ROC AUC score—Epitopes’ expression and nodal involvement

Epitope	AUC	Epitope	AUC	Epitope	AUC
CD1c	0.519	CD25	0.532	CD63	0.653
CD2	0.418	CD29	0.647	CD69	0.699
CD3	0.424	CD31	0.699	CD81	0.493
CD4	0.575	CD40	0.800	CD86	0.634
CD8	0.588	CD41b	0.630	CD105	0.619
CD9	0.681	CD42a	0.567	CD133	0.563
CD11c	0.551	CD44	0.647	CD142	0.652
CD14	0.584	CD45	0.467	CD146	0.618
CD19	0.649	CD49	0.659	CD209	0.615
CD20	0.616	CD56	0.466	CD326	0.677
CD24	0.665	CD62	0.641		

Moreover, we employed the Elastic Network model analysis as just proof for our findings and to strengthen statistical relevance. This is a multivariate regression analysis and one of the best statistical methods to infer the performance of combined predictors, based on L1 lasso and L2 ridge regularization. Nevertheless, our idea was to indicate practical biomarkers to be detected in the bloodstream using a comparative biosensor aligned and weighted on “normal” values, which is one of our future perspectives technologically and easier to obtain. In this light, we were thinking of a translational approach based on upregulated biomarkers that correlate to the specific clinical outcome, and, therefore, we would have excluded from the translational signatures also negatively correlating epitopes (even after bivariate analyses). This is the reason why we did not use this method as a first approach, knowing that it was likely to detect both epitopes overexpression and downregulation in combination as predictors for the signatures. Anyway, the obtained results were encouraging, corroborating our findings. In particular, CD2 and CD56 were selected by the model with a coefficient of 0.083 and 0.176, respectively, as positive predictors for tumor occurrence. CD40 obtained a 0.243 score, showing a remarkable capability to predict nodal involvement.

## Discussion

### Exosomal epitopes as biomarkers: state of art

In recent years, several studies have been centered on the identification of exosome-related biomarkers for disease diagnosis and prognosis. Such studies mostly focus on the analysis of exosome content or changes in number or size in certain pathologic conditions [3]. There are only few studies focusing on exosomal epitopes as disease biomarkers. Concerning the cancer research field, increased levels of CD105 have been found on extracellular vesicles’ surface of metastatic breast cancer patients through a multiplex assay aimed at detecting thirty-seven epitopes relevant in tumor-related immune response [15]. Recently, specific exosomal surface epitopes’ patterns were described for different subgroups of polytrauma patients, revealing the decrease of CD42+ exosomes regardless to the injury type and the modulation of other epitopes specifically related to the injury pattern [16]. Herein we unveil for the first time two carcer-relevant exosome epitope signatures linked either to tumor onset or to lymph node involvement.

### Interpretation of exosomal marker overexpression

The study’s analysis underscores the complexity of LCa’s derived exosome landscape. The overexpression in LCa patients of serum-derived exosomal epitopes correlating with cancer presence, compared to healthy controls (CD1c, CD2, CD3, CD4, CD14, CD11c, CD20, CD44, CD56, CD105, CD146, and CD209), alongside with specific ones linked to nodal involvement (CD24, CD31, CD40), suggests a multifaceted role for these markers in LCa pathology. To clarify the meaning of our findings, we thought it appropriate and important to recapitulate the epitopes’ role in order to drive the readers to the conclusion. In particular, the Tumor Presence Signature (TPS) herein described shows an AUC of 0.788 resulting from cumulative ROC curve analysis involving CD2, CD3, CD56 and CD146, thus delineating their common involvement in LCa cancer onset and progression. Exosomes expressing those epitopes are known for being excreted into the bloodstream by T-cells (CD2 and CD3)[17], NK-cells (CD56) [17] and cancer cells (CD146)[18]. Immune cells-derived exosomes might represent the high presence of those cells in patients indicating the chronic inflammation status triggered by LCa, despite being incapable of actively fighting the tumor and exerting their function in suppressing the cancer cells. CD146, on the other hand, is expressed by aggressive cancer cells and contributes to almost every step of the development and cancer progression, also serving as a receptor for growth factors and mediating vascular adhesion. The Nodal Involvement Signature (NIS) comprises CD24, CD31 and CD40 with a combined ROC curve AUC of 0.827 referring to nodal involvement. CD24 is a small adhesion protein implicated in tumor immune evasion, cancer aggressiveness and remarkably, also into epithelial to mesenchymal transition (EMT) [19, 20]. CD31 has been recognized as a cancer migration marker contributing to the invasiveness and associated with bad prognosis in different cancer subtypes [21]. CD40 has a less straightforward meaning in our setting. It is usually correlated to dendritic cells’ activation [17] and consequently to the enhancement of anti-tumor activity. In our specific case, it could be linked to tumor immune evasion which is a clear phenomenon also considering the TPS signature meaning, as well. Their association with immune response, cell adhesion, cancer growth and spreading indicates potential mechanisms through which LCa influences the tumor microenvironment and evades immune surveillance. The correlation of CD24, CD31, and CD40 with nodal involvement further emphasizes the potential of exosomal markers to reflect disease severity and metastatic potential.

### Challenges in exosomal research and clinical translation

The clinical translation of these findings faces several challenges, including the need for standardized exosome isolation and profiling techniques to ensure reproducible and accurate data. Moreover, while our study’s findings are promising and the patient cohort is remarkably higher than the average literature works on the subject, they necessitate validation in larger, more diverse cohorts to affirm their diagnostic value and understand their role in LCa’s biological landscape.

### Future directions and clinical implications

Our future research aims to elucidate the biological significance of TPS and NIS on LCa-derived exosomes. Understanding the pathways and interactions mediated by these markers and the reasons for their overexpression in body fluids could be crucial in the discovery of new therapeutic targets, especially from a liquid biopsy point of view. Moreover, obtaining valuable information on the actual content of those nanovescicles (DNA, RNA and Proteins) could actively explain and clarify the downstream pathway modulations. Additionally, the development of a standardized, sensitive, and non-invasive test based on these exosomal markers could drastically improve early LCa detection, facilitating timely intervention and improving prognosis. This is a crucial point for us, and in the next future one of our main goals will be to design and develop specific nanosensors based on overexpressed tumor exosomes for the point-of-care early diagnosis of the disease.

## Conclusion

Our study identified unique exosomal epitope signatures characterizing LCa patients. In particular, TPS can be exploited to differentiate LCa patients from healthy individuals allowing us an early detection only using patients’ peripheral blood withdrawal. The second signature identified, the NIS, could impact the discrimination of the patient prognosis indicating occult lymph node involvement, thus highlighting its potential for early disease severity assessment, and it is worth mentioning that there is, to date, no similar characterization about LCa in the published literature. Those findings are crucial for the integration of exosomal profiling into the diagnostic flow, promising to enhance early detection capabilities and, consequently, patient outcomes in laryngeal cancer. As we advance, the focus should be on overcoming the technical and validation challenges, aiming to bring this promising diagnostic tool from bench to bedside, unlocking new possibilities in the management and treatment of laryngeal cancer.

## Data Availability

The datasets generated and/or analyzed during the current study are available from the corresponding authors on request.
